# Resilience Story of Managing Severe Obstructive Sleep Apnea With Hypoventilation Secondary to SELENON (SEPN1)‐Related Myopathy

**DOI:** 10.1002/rcr2.70327

**Published:** 2025-08-27

**Authors:** Raghad Alhajaji, Hamza O. Dhafar, Muslim Mohammed Al Saadi, Ahmed S. BaHammam

**Affiliations:** ^1^ Sleep Medicine Fellowship King Saud University Medical City Riyadh Saudi Arabia; ^2^ Department of Medicine, College of Medicine, University Sleep Disorders Center King Saud University Riyadh Saudi Arabia; ^3^ Pediatrics Department, College of Medicine, Pediatric Pulmonology and Sleep Medicine, University Sleep Disorders Center King Khalid University Hospital, King Saud University Riyadh Saudi Arabia

**Keywords:** bilevel positive airway pressure (BPAP‐ST), REM‐related hypopnea, selenoprotein N, sleep‐disordered breathing, volume‐targeted pressure support (VtPS)

## Abstract

SELENON‐related myopathy is a rare autosomal recessive disorder characterised predominantly by muscle weakness; sleep‐disordered breathing and respiratory failure are frequent complications. We report a 15‐year‐old male with genetically confirmed SELENON‐RM and a 12‐year history of progressive sleep‐related breathing disorders, managed with overnight volume‐targeted pressure support (VtPS) positive airway pressure (PAP) plus supplemental oxygen. Longitudinal polysomnography (PSG) revealed evolution from mild REM‐predominant hypopneas to severe obstructive sleep apnea (OSA) with hypoventilation. Despite the elimination of apneas and hypopneas on bilevel positive airway pressure with spontaneous‐timed mode (BPAP‐ST), persistent desaturation occurred during REM sleep. Transitioning to VtPS nocturnal PAP combined with oxygen‐corrected hypoventilation normalised oxygen saturation across all sleep stages and abolished respiratory events.

## Graphical abstract

The treatment response to overnight volume‐targeted pressure support (VtPS), positive airway pressure plus supplemental oxygen, dramatically improved and eliminated respiratory events while maintaining oxygen saturation compared to bilevel positive airway pressure in spontaneous/timed (BPAP‐ST) mode in cases with severe obstructive sleep apnea (OSA) with hypoventilation secondary to neuromuscular disorders. The case report and treatment are detailed here.

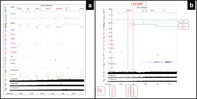

## 1 Introduction

SELENON‐related myopathy (previously known as SEPN1‐RM), or multiminicore disease, is a congenital myopathy characterised by multiple small core lesions in the muscle fibres. It is associated with mutations in SEPN1, which encodes selenoprotein N1, leading to various myopathic changes [1, 2, 3]. Disease onset typically appears within the first 2 years of life [1]. Clinical presentations include muscle hypotonia, delayed motor development, and respiratory impairment, with spinal rigidity and scoliosis being prominent features [1, 2, 3]. Diagnosis involves clinical evaluation, genetic testing, and muscle biopsy, with muscle MRI aiding in the identification of distinct patterns [4].

A retrospective case series showed that 85% of cases present within the first 2 years of life [5]. Although early motor milestones may be achieved, respiratory involvement is nearly universal, affecting 94% of patients, with 93% developing restrictive respiratory failure. Reduced forced vital capacity (FVC) is common, with 85% of patients showing FVC below 50% [5, 6]. Additionally, 93% of patients had nocturnal hypoventilation, and approximately 82% required assisted ventilation [5].

Three reported cases had onset between the ages of 7 and 15 years, with PSG revealing predominantly rapid eye movement (REM)‐related respiratory events and nocturnal hypercapnia [6]. Although sleep‐disordered breathing is a well‐recognised complication of SELENON‐related myopathy, the relationship between its onset and disease progression remains poorly understood. Furthermore, there is no established consensus on the optimal ventilatory support strategies, as existing approaches vary and often require individualised adjustments based on disease severity and polysomnographic findings [6, 7].

Sleep‐disordered breathing in SELENON‐RM can emerge early, yet its presentation, progression, and optimal management remain poorly understood. This case report presents unique longitudinal polysomnographic data spanning 12 years, illustrating the progression from mild REM‐related hypopnea to severe OSA with hypoventilation.

## 2 Case Report

### 2.1 Early Signs and Diagnosis

A 15‐year‐old male child born to consanguineous parents (second‐degree cousins) presented at 18 months with delayed gross‐motor milestones and nocturnal mouth breathing. Physical examination showed axial hypotonia and mild scoliosis. Electromyography and muscle biopsy results were consistent with multiminicore myopathy; subsequent exome sequencing identified biallelic pathogenic variants in SELENON, confirming SELENON‐related myopathy (SELENON‐RM). Three paternal cousins exhibited a similar presentation, and two died of respiratory failure, but genotyping was unavailable.

### 2.2 Diagnostic Study Findings

By the age of 2.5 years, the patient's first attended PSG showed mild REM‐related hypopneas. The patient slept for a total sleep time (TST) of 6 h and 30 min with an apnea Hypopnea index (AHI) of 3.5 events per hour (NREM/REM) (1.8/16.9). REM duration was 42.5 min, with a desaturation index of 7.5/h and the lowest saturation of 85%.

Serial PSGs captured stepwise deterioration. In 2016 (age 7), a repeat study, prompted by parental reports of louder snoring, revealed moderate obstructive sleep apnea (OSA) with intermittent saturations to 78%. Cardiorespiratory status remained stable until 2021, when overnight PSG showed severe REM‐related OSA with hypoventilation: AHI 32.4/hr.; EtCO_2_ > 55 mmHg for 87 min while awake, 155 min during NREM, and 29 min during REM (peaks 69 and 65 mmHg, respectively). SpO_2_ fell repeatedly to 40% with arousals (Table 1).

**TABLE 1:** Main finding summary of the sleep studies.

Date of test	Type of test	CPAP/BPAP/O_2_	Age	AHI (NREM/REM)	Arousal index (/hr)	Lowest O_2_	Average O_2_	Desaturation index (/hr)
10‐Jun‐2012	Overnight sleep study	NR	3 years	3.5 (1.8/16.9)	6	85%	98%	7.5
16‐Oct‐2016	Overnight sleep study	NR	6 years	6.8 (1.8/31.1)	10.2	62%	97%	4.1
15‐Nov‐2021	Overnight sleep study	NR	11 years	32.4 (24.4/74.1)	8.4	40%	86%	52.1
31‐Jan‐2022	Overnight therapeutic sleep study	CPAP 8 cmH_2_O, BPAP 14/9 cmH_2_O, BPAP 18/12 cmH_2_O + O_2_ 1L	12 years	CPAP: 37.4 (37.4/−) BPAP: 29.7 (21.1/112.9) BPAP plus O_2_: 18.9 (9.9/38.4)	CPAP: 1.6 BPAP: 5.3 BPAP + O_2_: 3.9	CPAP: 58% BPAP: 39% BPAP + O_2_: 87%	CPAP and BPAP: 86% BPAP + O_2_: 97%	CPAP: 60.8 BPAP: 22.4 BPAP + O_2_: 0.8
26‐Dec‐2022	Overnight therapeutic sleep study	BPAP 20/16 cmH_2_O + O_2_ 1 LPM	12 years	27.9 (13.8/83.8)	N/A	48%	94%	38
23‐Oct‐2024	Daytime therapeutic sleep study	BPAP ST 20/12 cmH_2_O + O_2_ 3 LPM	14 years	14.8 (3.8/36.6)	16.3	20%	86%	43
28‐Nov‐2024	Daytime therapeutic sleep study	AVAPS‐AE + O_2_ 0.5 LPM	14 years	0.7 (0.3/2.5)	7.4	91%	96%	6.5

Abbreviations: AHI, Apnea‐Hypopnea index; Arousal index (/hr); the number of arousals or awakenings per hour of sleep; AVAPS‐AE, average volume assured pressure support—auto‐expiratory; BPAP ST, Bi‐level positive airway pressure with spontaneous/timed mode; BPAP, Bi‐level positive airway pressure; CPAP, continuous positive airway pressure; Desaturation index (/hr), the number of desaturation events (drops in oxygen saturation) per hour of sleep; EPAP, expiratory positive airway pressure; Lowest O_2_/average O_2_, lowest and average oxygen saturation levels during sleep; LPM, litres per minute; Max, maximum; Min, minimum; NR, not reported; NREM, non‐rapid eye movement; O_2_, oxygen; PS, pressure support; REM, rapid eye movement; RR, respiratory rate; TcCO_2_, carbon dioxide measured through the skin; Tidal volume, the volume of air inhaled or exhaled during each breath.

Pulmonary function mirrored sleep findings. Between 2018 and 2024, FVC declined from 0.80 L (41% predicted) to 0.33 L (17%); forced expiratory volume in 1 s (FVC1) from 0.74 to 0.32 L. The FEV_1_/FVC ratio remained > 0.93, confirming a restrictive pattern; total lung capacity decreased from 1.98 to 1.09 L.

### 2.3 Therapeutic Challenges

The patient's condition has deteriorated since 2020; he started BPAP with pressures of 15/−4 cm of water pressure (cmH_2_O) and later changed to 12/6 cmH_2_O.

**FIGURE 1:**
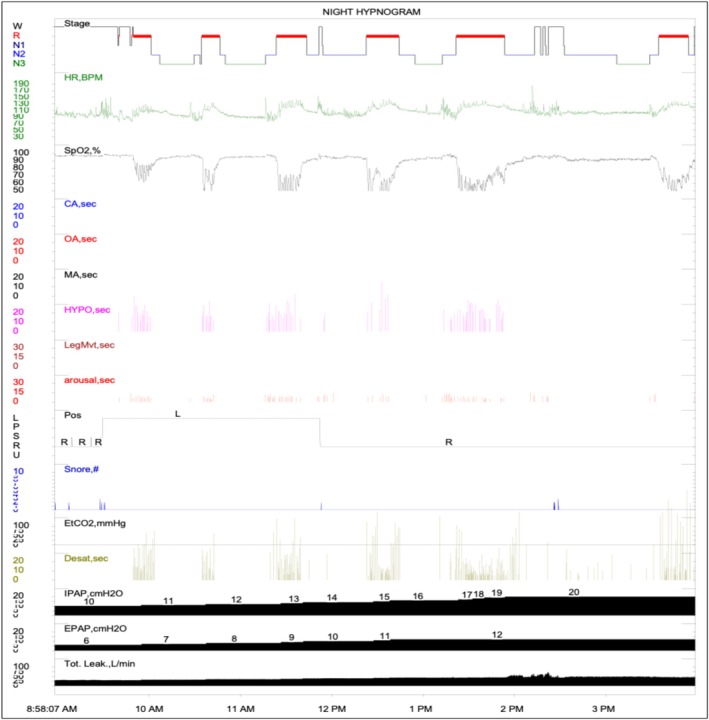
Histogram of therapeutic bilevel positive airway pressure in spontaneous/timed (BPAP‐ST) plus oxygen therapy sleep study.

On October 23, 2024, a therapeutic study was conducted using BPAP ST at 20/12 cmH_2_O with oxygen at 3 L per minute (LPM). Obstructive hypopneas were eliminated in both REM and non‐REM sleep, while desaturation was eliminated in lateral NREM sleep. However, sustained desaturation persisted during REM sleep, despite the patient receiving 3 LPM of oxygen. The AHI during this study was 14.8 (3.8/36.6), and the lowest recorded oxygen saturation was 20%. Transcutaneous Carbon Dioxide (TcCO_2_) measurements ranged from 84 to 100 mmHg during NREM sleep and from 62 to 100 mmHg during REM sleep (Figure 1).

The decision to transition to VtPS was based on the need to address both persistent hypoventilation and oxygen desaturation, despite the resolution of obstructive events. Given that sustained hypoxia persisted, VtPS was selected to optimise ventilation by ensuring a consistent tidal volume and improving oxygenation.

**FIGURE 2:**
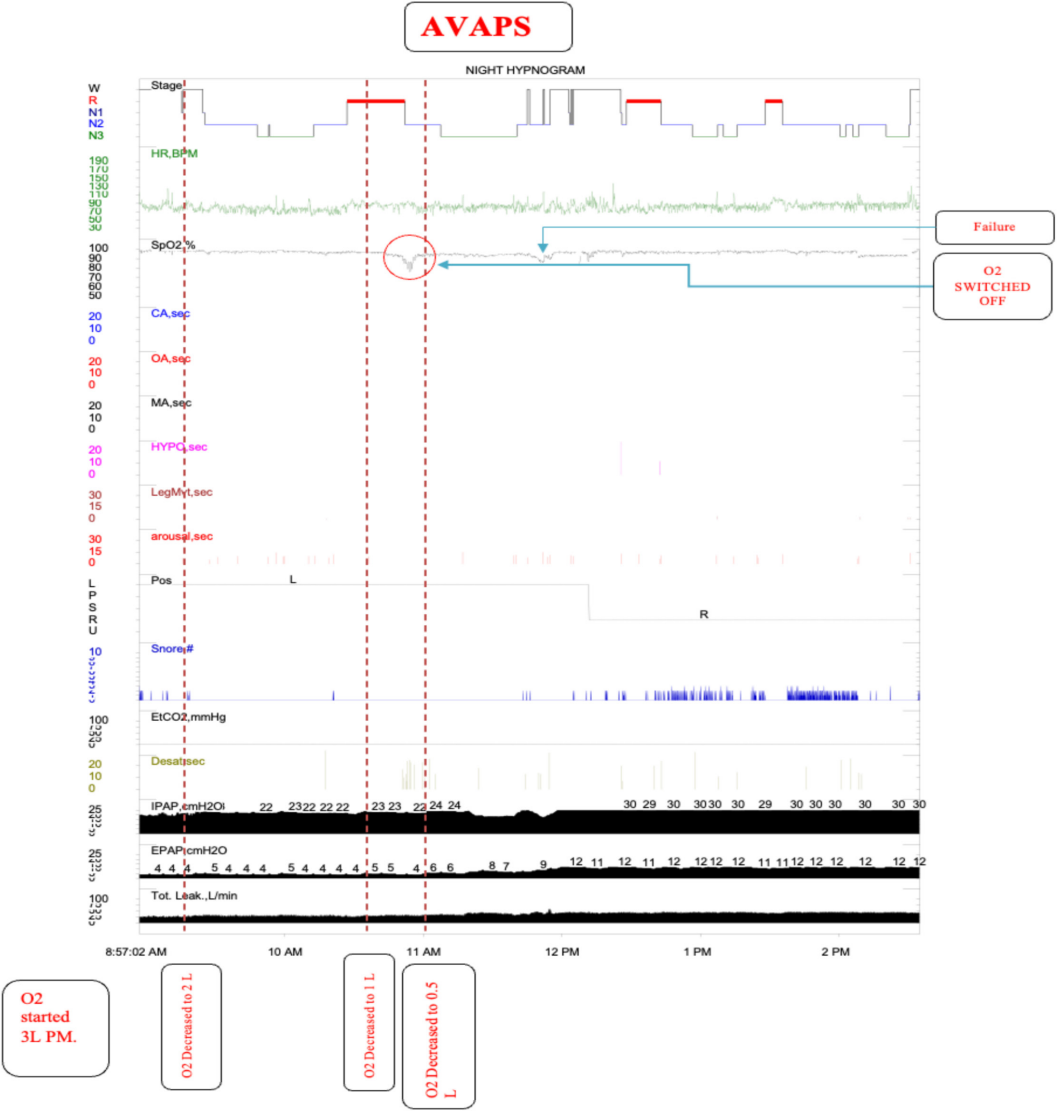
Histogram of therapeutic on volume‐targeted pressure support (VtPS) nocturnal PAP plus oxygen therapy sleep study. Arous, Arousals; AVAPS, average‐volume‐assured pressure support; BPM, beats per minute; CA, central apnoea; cmH_2_O, centimetres of water (pressure unit); Desat, oxygen desaturation; EPAP, expiratory positive airway pressure; EtCO_2_, end‐tidal carbon‐dioxide; HR, heart rate; HYP, hypopnoea; IPAP, inspiratory positive airway pressure; L/min, litres per minute (flow‐rate unit); LegMvt, leg movements; MA, mixed apnoea; N1/N2/N3, non‐REM sleep stages 1, 2 and 3; O_2_, supplemental oxygen; OA, obstructive apnoea; Pos, body position (L = Left, *P* = Prone, S = Supine, *R* = Right, U = Unknown); R, REM (rapid‐eye‐movement) sleep stage; Snore, snoring events counter; SpO_2_, peripheral oxygen saturation; Tot. Leak, total mask/system leak; W, wake stage.

Standard pressure support may not have adequately addressed the residual hypoventilation, as it provides fixed pressure support without adjusting for changes in ventilation needs. VtPS was chosen because it gradually adapts pressure support to maintain a consistent tidal volume, thereby improving ventilation and oxygenation. Consequently, on November 28, 2024, we moved to another therapeutic study that was performed using VtPS with oxygen at 0.5 LPM. The device settings were Min EPAP = 4 cmH_2_O, Max EPAP = 12 cmH_2_O, Min PS = 8 cmH_2_O, Max PS = 18 cmH_2_O, Tidal Volume = 300 mL, Max Pressure = 30 cmH_2_O, Breath Rate: Auto, and VtPS Rate: 2 cmH_2_O/min. This study demonstrated the successful elimination of respiratory events during lateral REM sleep, with a significant improvement in oxygen saturation and sustained hypoxia. The lowest oxygen saturation level was 91%, the average was 96%, and the desaturation index was 6.5. The AHI was 0.7 (0.3/2.5) (Table 2). The VtPS provided successful management of the patient's hypopneas and hypoventilation during sleep, with notable improvements in oxygen saturation levels compared to previous therapeutic approaches, with the lowest O_2_ of 91%. Figure 2 illustrates the corresponding sleep study results. Significant subjective and objective improvements were reported upon regular follow‐up.

**TABLE 2:** Comparison between the effectiveness of therapeutic titration between BPAP‐ST and VtPS mode.

BPAP‐ST mode	AVAPS‐AE mode
The pressure of 20/12 cmH_2_O with Oxygen 3 LPM, respiratory events were eliminated during lateral NREM sleep; however, they still persisted in REM sleep with sustained desaturation. In the last REM cycle with the BPAP pressure of 20/12 cmH_2_O, sustained desaturation was noted without respiratory events. He progressed into all sleep stages during the study. Otherwise, no unusual events were noted. TcCO_2_ values showed: 30–100 mmHg while awake. 84–100 mmHg in NREM sleep. 62–100 mmHg in REM sleep.	The patient slept for a total sleep time of 4 h and 36.5 min. Titration reached AVAPS‐AE settings: Min EPAP: 4 cmH_2_O Max EPAP: 12 cmH_2_O Min PS: 8 cmH_2_O Max PS: 18 cmH_2_O Tidal volume: 300 mL Max pressure: 30 cmH_2_O Breath rate: Auto AVAPS rate: 2 cmH_2_O/min Oxygen: 0.5 LPM Respiratory events were eliminated during lateral REM sleep, and oxygen saturation significantly improved as well. He progressed into all sleep stages during the study.

Abbreviations: AVAPS‐AE, average volume‐assured pressure support—Auto EPAP mode; BPAP‐ST, bilevel positive airway pressure—spontaneous timed mode; cmH_2_O, centimetres of water pressure (used for respiratory device settings); EPAP, expiratory positive airway pressure; LPM, litres per minute (oxygen flow rate); NREM, non‐rapid eye movement (sleep stage); PS, pressure support; REM, rapid eye movement sleep stage; TcCO_2_, transcutaneous carbon dioxide.

## 3 Discussion

This longitudinal case report documents the progressive decline in respiratory function over 12 years in a patient with SELENON‐related myopathy (SELENON‐RM). The patient's journey illustrates several key aspects of this rare condition's natural history and management challenges.

The pulmonary function tests revealed a dramatic deterioration, with FVC decreasing from 0.80 L in 2018 to just 0.33 L in 2024, alongside a parallel reduction in FEV1 from 0.74 to 0.32 L. This severe restrictive pattern exceeds the typical decline reported in the literature, where average FVC values hover around 52% of predicted [7]. The patient's total lung capacity (TLC) similarly dropped from 1.98 to 1.09 L, while the relatively stable residual volume/total lung capacity (RV/TLC) ratio (68%–65%) suggests ongoing air trapping and progressive respiratory muscle weakness.

Sleep‐disordered breathing in this patient evolved from mild REM‐related hypopneas in 2012 to severe OSA with hypoventilation by 2021, mirroring the pattern described in previous case reports [6]. This progression necessitated increasingly sophisticated ventilatory support. Initial management with BPAP at 15/−4 cmH_2_O, later adjusted to 12/6 cmH_2_O, became insufficient as the disease advanced. By early 2022, even BPAP at 18/12 cmH_2_O with 1 LPM oxygen supplementation only partially controlled respiratory events (AHI 18.9) without fully resolving hypoxemia.

The most striking finding was the persistent REM‐related desaturations despite escalation to BPAP S/T at 20/16 cmH_2_O with oxygen increased to 3 LPM. During REM sleep, oxygen saturation plummeted to as low as 20%, highlighting the limitations of conventional pressure‐targeted ventilation in neuromuscular disorders with variable respiratory drive and mechanics across sleep stages.

The transition to VtPS therapy marked a turning point in management. This advanced mode, which combines volume targeting with auto‐adjusting expiratory pressure, effectively eliminated respiratory events during all sleep stages and dramatically improved oxygenation (lowest SpO_2_ 91%). The success of VtPS in this case suggests that volume‐targeted ventilation may be particularly beneficial in neuromuscular disorders with sleep‐state‐dependent respiratory compromise.

While positive airway pressure therapy remains the gold standard for OSA management [8], recent technological advances have significantly enhanced PAP capabilities. Modern ventilatory modes featuring auto‐titrating algorithms, adaptive servo‐ventilation, and volume‐targeted pressure support systems offer more personalised approaches to complex sleep‐disordered breathing patterns [8]. Our case demonstrates the practical application of these innovations in managing a challenging neuromuscular condition.

Previous reports have documented that nocturnal hypoventilation in SELENON‐RM typically worsens over time, especially during REM sleep, often necessitating noninvasive ventilation (NIV) in S/T mode [6, 9].

Our patient's experience aligns with these observations but further illustrates that conventional BPAP S/T may be insufficient for some patients, particularly during REM sleep. While some cases require tracheostomy when NIV fails to control hypercapnia [8], our patient achieved adequate ventilation with VtPS, avoiding more invasive interventions.

In conclusion, this case report provides unique longitudinal data documenting the progression of sleep‐disordered breathing in SELENON‐RM over 12 years. Our findings highlight a critical limitation of conventional BPAP‐ST therapy: persistent REM sleep desaturation despite elimination of visible respiratory events. The successful implementation of VtPS therapy—which improved oxygen saturation, eliminated respiratory events, and maintained effective ventilation across all sleep stages—suggests that volume‐targeted ventilation may be superior to pressure‐targeted modes in managing complex sleep‐disordered breathing in neuromuscular disorders.

## Author Contributions

Raghad Alhajaji conceived the case report, obtained patient consent, collated longitudinal clinical and polysomnographic data, drafted the manuscript, and prepared all figures and tables. Hamza O. Dhafar supervised sleep‐study acquisition and titration, interpreted respiratory signals, and performed the critical revision of the text for scientific accuracy. Ahmed S. BaHammam and Muslim Mohammed Al Saadi provided senior oversight, contributed expertise in neuromuscular sleep disorders, refined the clinical narrative, reviewed and revised, and gave final approval of the version to be submitted. All authors fulfil the ICMJE authorship criteria and agree on the final version of the submission.

## Consent

The authors declare that written informed consent was obtained for the publication of this manuscript and accompanying images and attest that the form used to obtain consent from the patient complies with the journal requirements as outlined in the author guidelines.

## Conflicts of Interest

The authors declare no conflicts of interest.

## Data Availability

The data that support the findings of this study are available on request from the corresponding author. The data are not publicly available due to privacy or ethical restrictions.
